# Direct monitoring of trace water in Li-ion batteries using *operando* fluorescence spectroscopy†
†Electronic supplementary information (ESI) available. See DOI: 10.1039/c7sc03191b


**DOI:** 10.1039/c7sc03191b

**Published:** 2017-10-23

**Authors:** Xiaoyan Ren, Jiawei Wang, Zhangquan Peng, Lehui Lu

**Affiliations:** a State Key Laboratory of Electroanalytical Chemistry , Changchun Institute of Applied Chemistry , Chinese Academy of Sciences , Changchun 130022 , China . Email: zqpeng@ciac.ac.cn ; Email: lehuilu@ciac.ac.cn

## Abstract

The direct monitoring of trace water in real time during electrochemical cycles is of vital importance because water impurities are one of the causes of reduced lifetimes and capacity fading in Li-ion batteries. However, the most common Karl-Fischer titration lacks the ability to perform real-time monitoring of trace water while the battery is operating. Here, we demonstrate the use of nanosized coordination polymers as a sensing platform for the rapid and highly sensitive detection of water molecules, which gives a distinguishable turn-on fluorescence (FL) response toward water with a quantifiable detection range from 0 to 1.2% v/v, offering a novel opportunity to monitor trace water during electrochemical cycles. To demonstrate the practical value of our platform, we designed an *in situ* measurement system using nanosized coordination polymers as an electrolyte additive. Within the platform, the findings indicate that trace water is indeed generated during the first discharge process, in which the FL intensity shows a linear increase over time along with the gradual formation of water. We believe that this strategy provides new insights into the *in situ* monitoring of complex electrochemical processes, and it may help to pave the way for the development of new *operando* analytical techniques for lithium-ion batteries.

## Abstract 2

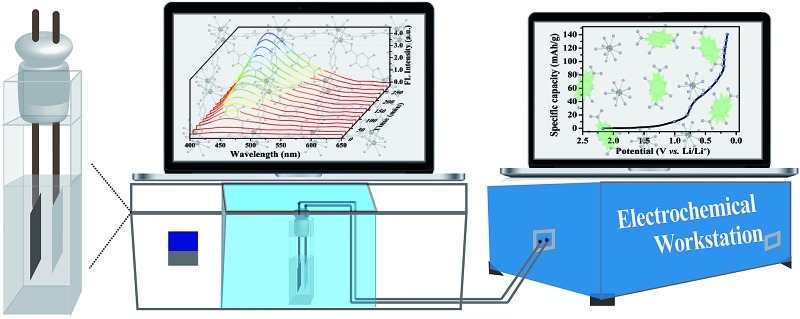
 *Operando* fluorescence spectroscopy provides an effective platform for the direct monitoring of trace water in an operating Li-ion battery, with the assistance of nanosized coordination polymers as fluorescent probes.

## Introduction

During the past few decades, lithium-ion batteries have been considered as the most promising energy storage devices due to their outstanding features of high power density, high energy density, long cycle life and environmental friendliness.^1^,^2^ However, the reduced lifetime and capacity fading still represent a major challenging issue.^3^,^4^ One of the possible reasons for this fading phenomenon is the presence of water impurities which are unavoidably present in the liquid electrolytes or are caused by side reactions during the electrochemical cycles.^5^,^6^ As is well-known, water impurities have significant negative effects on battery performances, and they cause poor cycling performance and the loss of active materials. Firstly, water can react with active lithium foils and the common electrolyte, LiPF_6_, thus resulting in capacity fading.^7^,^8^ Secondly, water can destroy the protective solid electrolyte interface (SEI) layer, which works as a kind of passivation layer to protect the electrodes’ active components, and prevents electrolyte degradation by resisting electron transport and allowing lithium ions to pass through.^9^,^10^ Moreover, water can be reduced on the anode to yield hydrogen gas, increasing the internal pressure of the battery, which will further bring about potential safety hazards.^11^,^12^ Therefore, it is increasingly essential to develop an innovative technique to directly monitor water impurities in Li-ion batteries, and furthermore to provide molecule-level insights into understanding water formation and thereby improving battery performances. Nevertheless, the real-time monitoring of water content in a working battery is limited by the difficulties of *operando* measurements. The most common off-line electrochemical method, Karl-Fischer titration, fails to track the water content while the battery is operating.^13^ Even though many *operando* analytical techniques, such as NMR,^14^,^15^ EPR,^16^ TEM^17^ and X-ray diffraction,^18^ have been operated to monitor complex electrochemical processes,^19^–^21^ none of these approaches are able to directly measure the water content in liquid electrolytes during electrochemical cycling. To this end, there is an urgent demand for new *operando* analytical techniques that can realize the direct monitoring of trace water in an operating Li-ion battery.

Luminescent probes show significant advantages, including the advantages of *in situ* measurements, ease of fabrication, low cost, short acquisition time and naked-eye detection.^22^,^23^ So far, most water probes are limited by the usage of organic fluorescent molecules and quantum dots, which are applied for water detection in organic solvents.^24^,^25^ However, the majority of these probes are incapable of detecting H_2_O within an electrolyte containing ppm levels of water. Given these circumstances, novel water probes with ultra-sensitivity are highly desired to realize the direct monitoring of trace water in organic solvents, and especially in working batteries. Herein, we report the direct monitoring of trace water in a Li-ion cell during operation using *operando* fluorescence spectroscopy. To demonstrate the practical value of our platform, we designed an *in situ* measurement system using nanosized coordination polymers as an electrolyte additive, which gives a distinguishable “turn-on” fluorescence response toward water with a quantifiable detection range from 0 to 1.2% v/v. Within the platform, the results indicate that trace water is indeed generated during the first discharge process, in which the FL intensity shows a linear increase over time along with the gradual formation of water. Our approach provides a novel strategy for the quantitative measurement of water content and the *in situ* tracking of complex electrochemical processes.

## Experimental

### Materials and reagents

2,5-Dihydroxyterephthalic acid (DHBDC), acetonitrile (MeCN), dimethyl formamide (DMF) and anhydrous terbium chloride were purchased from Sinopharm Chemical Reagent Co., Ltd. SFG-15 graphite powder (Timcal Co. Ltd.) and polytetrafluoroethylene (PTFE, Aldrich) were used as received. All organic solvents and electrolytes, including ethyl methyl carbonate (EMC), ethylene carbonate (EC) and diethyl carbonate (DEC), were of analytical grade and were dried using 4 Å molecular sieves (NaA zeolite) until the H_2_O concentration was lower than 10 ppm, determined by a Mettler-Toledo Karl-Fischer titration apparatus.

### Synthetic procedures

#### Preparation of the bulk crystals

A mixture of TbCl_3_ (0.135 g) and 2,5-dihydroxy-terephthalic acid (DHBDC, 0.099 g) was dissolved in 10 mL DMF, then loaded into a stainless steel vessel (20 mL). The vessel was sealed and heated to 60 °C for two days. After the reaction mixture was cooled down to room temperature, plate-like crystals were obtained, which were further washed with DMF three times.

#### Preparation of the nanoprobe

The nanosized product was successfully synthesized on a large-scale *via* a nano-precipitation process. TbCl_3_ (26.5 mg) was mixed with 2,5-dihydroxyterephthalic acid (DHBDC, 20.0 mg) in DMF (5.0 mL) solution and heated at 60 °C for two hours. Afterwards, MeCN was added quickly to form the nanosized precipitate (*V*_DMF_/*V*_MeCN_ = 1 : 2). The precipitate was collected by centrifugation, washed with MeCN several times, then redispersed in MeCN. Elemental analysis: measured: Tb, 24.25; C, 36.84; H, 4.83 wt%; theoretical: Tb, 23.63; C, 37.48; H, 4.02 wt%. The UV-Vis spectrum, TGA results and FTIR analysis of the nanoprobe are displayed in Fig. S1–S3.†

### Electrochemical characterization

In order to realize the simultaneous analysis of the electrochemical properties and fluorescence spectra, an *in situ* cell was home-made with an external size of 2.2 × 2.2 × 4 cm^3^ and a suitable silica gel plug with two circular holes with diameters of 2 mm. The composite carbon electrodes were obtained by mixing 90 wt% SFG-15 graphite and 10 wt% polytetrafluoroethylene in 2-propanol to form uniform slurries, which were then coated onto a stainless steel mesh current collector. The prepared electrodes were dried under vacuum at 60 °C overnight. The loaded active material was about 2–3 mg cm^–2^ under the experimental conditions. As for the counter/reference electrode, a slice of lithium foil was pressed on a stainless steel mesh current collector. The electrolyte was 1 M LiPF_6_ in a mixture of 1EC : 1EMC : 1DEC. Stock electrolyte solutions were prepared by dispersing the freeze-dried nanoprobe in the above electrolyte with a concentration of 10 μg mL^–1^. The 2-electrode cell consisted of thin Li foil as the counter/reference electrode and a composite carbon electrode as the working electrode. The cell was assembled in an argon-filled glove box and finally sealed using parafilm to avoid contamination by moisture and oxygen. The electrochemical tests were performed on a Parstat 4000 electrochemical workstation with a voltage range of 0.01–3.0 V *vs.* Li^+^/Li. CR2032-type coin cells were assembled using a similar composite to the electrodes, and sealed in an argon-filled glove box to avoid external contamination. The electrochemical cycles were recorded on a LANHE CT2001C multi-channel battery testing system with a voltage range of 0.01–3.0 V *vs.* Li^+^/Li.

## Results and discussion

**Scheme 1:**
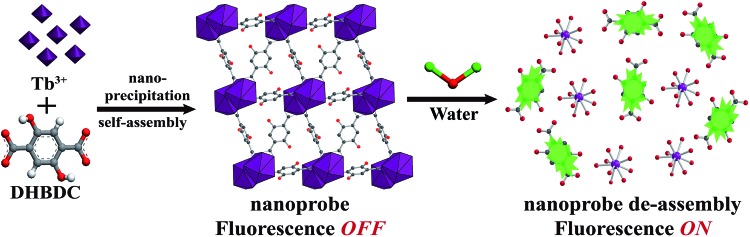
Schematic illustration of the formation and sensing processes of the nanoprobe. DHBDC = 2,5-dihydroxyterephthalic acid.

**Fig. 1:**
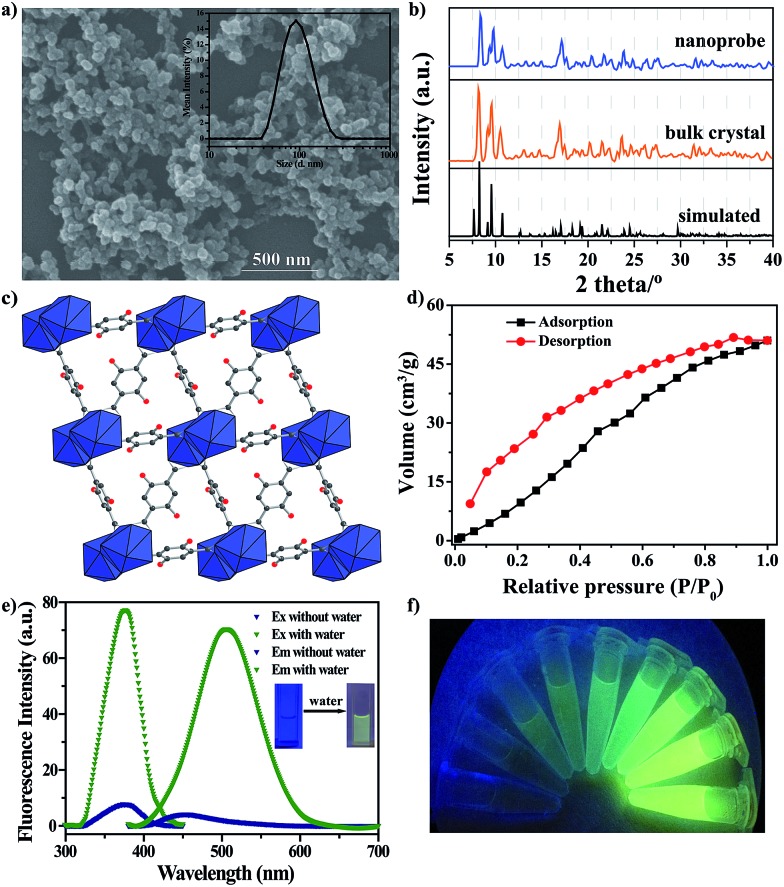
(a) SEM image. The inset shows the DLS analysis. (b) XRD patterns. (c) The framework. (d) CO_2_ adsorption/desorption isotherms obtained for the nanoprobe. (e) Fluorescence excitation (Ex) and emission (Em) spectra of the nanoprobe dispersion in dry MeCN without and with water. (f) Visible color changes of the nanoprobe in the presence of successive aliquots of water when irradiated with UV light of 365 nm. The water content was 0, 0.05, 0.5, 1, 2, 3, 4, 5 and 6% v/v.

The nanoprobe was synthesized on a large-scale *via* a nano-precipitation process, as shown in Scheme 1. The SEM image in Fig. 1a revealed that the nanoprobe was within an average size of *ca.* 100 nm, which was consistent with the dynamic light scattering (DLS) analysis (Fig. 1a inset). The XRD patterns of the nanoprobes were in accordance with those from the bulk crystals and the single crystal structure data (Fig. 1b). The structure was isomorphous to [La_2_(DHBDC)_3_(DMF)_4_] (DHBDC = 2,5-dihydroxyterephthalic acid, DMF = *N*,*N*-dimethylformamide),^26^ wherein each di-nuclear terbium unit was connected to six DHBDC ligands and four DMF molecules, and further linked together to form a 3-D structure (Fig. 1c, S4 and S5†). The nanoprobe could adsorb CO_2_ but exhibited nonporous behavior toward N_2_, thus demonstrating the absence of permanent channels in the intrinsic structure. As shown in Fig. 1d, the maximum CO_2_ uptake for the nanoprobe at 1 atm is 51.2 cm^3^ g^–1^, which is similar to that of the bulk crystals (55.7 cm^3^ g^–1^). The nanoprobe dispersion exhibited a weak turquoise fluorescence with a quantum yield of 0.32% when irradiated with UV light of 365 nm. Upon the addition of water with a volumetric ratio of 5%, the nanoprobe showed an intense yellowish emission with a quantum yield of 33.23%. Fig. 1e shows the excitation and emission spectra of the nanoprobe without and with water. The fluorescence intensity of the nanoprobe was enhanced by almost 40 times with the presence of water, accompanied by an obvious color change from weak turquoise to intense yellow, enabling naked-eye water detection (Fig. 1f).

**Fig. 2:**
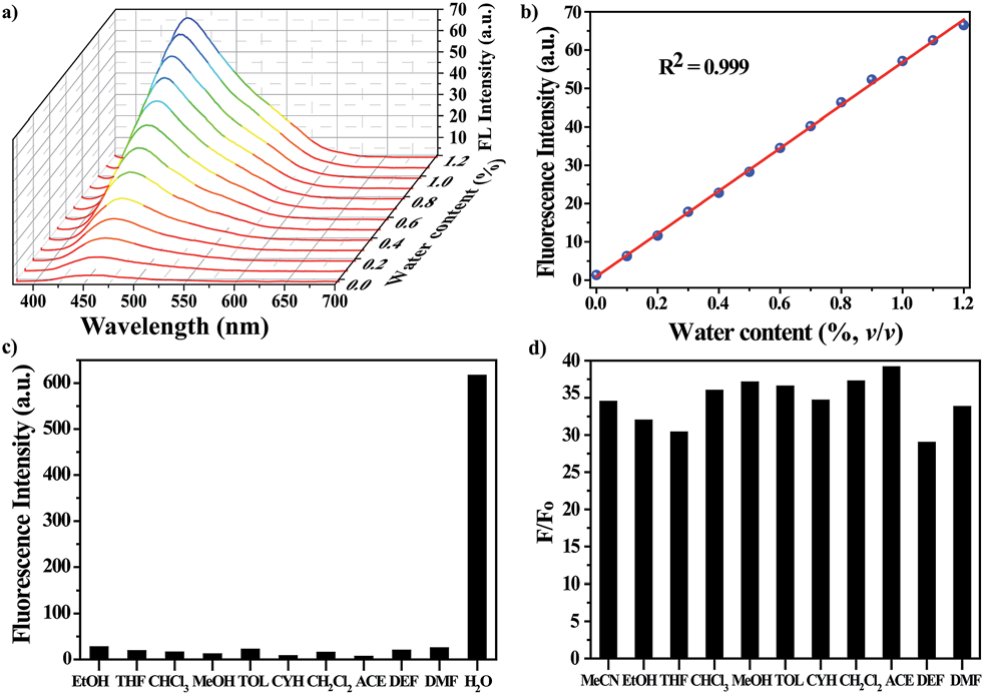
(a) Changes in the fluorescence spectra of the nanoprobe dispersion in dry MeCN in the presence of successive aliquots of water (0–1.2% v/v). The excitation wavelength was *λ*_ex_ = 360 nm. (b) Plot of the fluorescence intensity as a function of water content. (c) Selectivity of the nanoprobe for water over other representative organic solvents. (d) Plot of *F*/*F*_0_ in various organic solvents in the presence of water. *F*_0_ and *F* are the FL intensities of the nanoprobe in the absence and presence of water, respectively.

The water-responsive property of the nanoprobe prompted us to investigate its performance as a luminescent probe in organic solvents, taking dry acetonitrile (MeCN) as an example. The sensitivity of the nanoprobe was evaluated by monitoring the fluorescence changes with the introduction of successive amounts of water (Fig. 2a and S6†). The intensity of the fluorescence emission at 450 nm increased gradually upon increasing the water content. Fig. 2b reveals a good linear correlation describing the fluorescence intensity as a function of the water content over the range of 0–1.2% v/v (*R*^2^ = 0.999). The limit of detection (LOD) for water molecules at a signal-to-noise (S/N) ratio of 3 was determined to be 0.03%. Remarkably, all of the measurements were performed within one minute, suggesting that the nanoprobe displayed a fast response to water stimuli in organic solvents. These results showed that the nanoprobe could provide an effective detection platform capable of the sensitive detection of water molecules, and it gave a turn-on fluorescence response with a good linear correlation over a concentration range of 0–1.2% v/v.

Along with the sensitivity requirements, specificity is highly necessary for practical applications. For this purpose, we measured the fluorescence changes in the presence of organic solvents with a volumetric ratio of 5% (Fig. 2c), including ethanol (EtOH), tetrahydrofuran (THF), trichloromethane (CHCl_3_), methanol (MeOH), toluene (TOL), cyclohexane (CYH), dichloromethane (CH_2_Cl_2_), acetone (ACE), diethyl formamide (DEF) and *N*,*N*-dimethyl formamide (DMF). As expected, none of the above solvents induced any noticeable changes in the FL intensity of the nanoprobe, showing that the nanoprobe-based detection system is highly specific toward water. Besides MeCN, the nanoprobe could be also utilized to detect water molecules in other organic solvents, and it showed a similar turn-on FL response to water stimuli, where the FL intensity of the nanoprobe was largely enhanced with the addition of water (Fig. 2d).

**Fig. 3:**
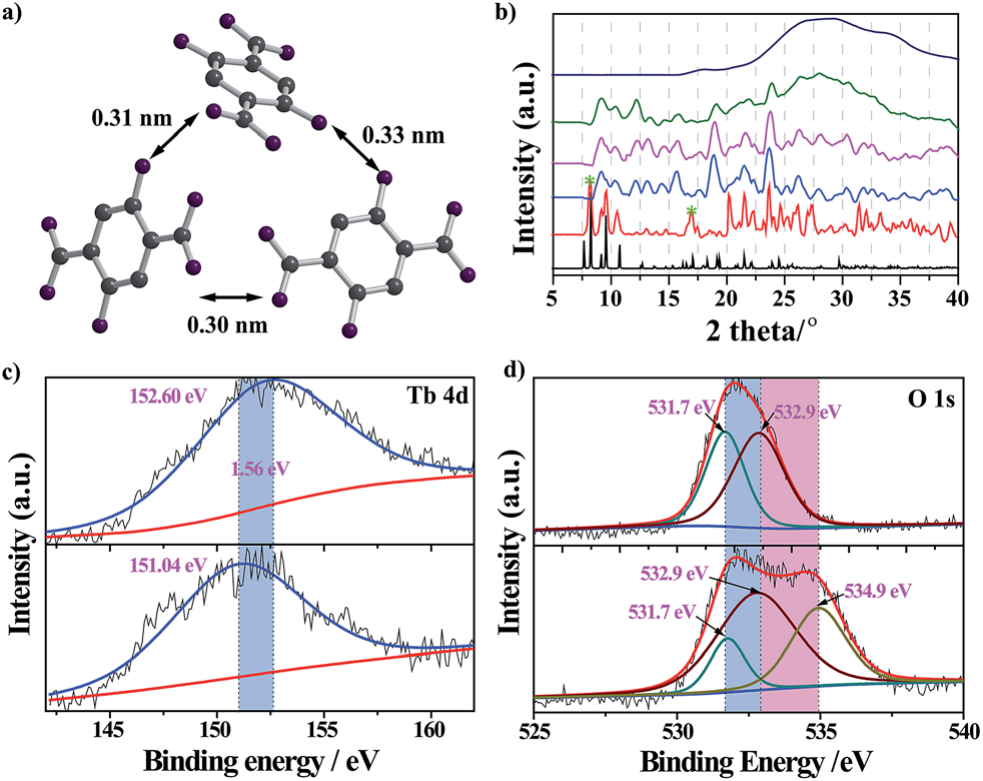
(a) The distance between DHBDC ligands in the nanoprobe. This distance is below the critical value for energy transfer, leading to fluorescence quenching. (b) The XRD patterns change with the induction of successive amounts of water. The water content is, from bottom to top: 0, 1, 5, 10, 25 and 50% v/v. (c) High resolution Tb4d and (d) O1s XPS spectra of (top) the nanoprobe without water and (bottom) the nanoprobe with water.

The nanoprobe itself exhibited quite weak fluorescence properties, possibly because of the self-quenching property of the DHBDC ligands. As discussed above, the absence of permanent micropores indicated that it possessed a relatively dense structure and that the H_2_DHBDC···H_2_DHBDC distances were small (Fig. 1c and d). The inter-ligand distance (0.32 nm on average) from the structural data analysis was below the critical radius for coulombic energy transfer (1–10 nm) as shown in Fig. 3a, and thus the self-quenching process of the DHBDC ligands occurred.^27^ Upon exposure to water, water molecules as competitive ligands began to coordinate with the trivalent Tb^3+^ ions, resulting in ligand displacement and structural decomposition. The DHBDC ligands were unbound to the Tb^3+^ ions and exhibited their intrinsic fluorescence. From the *operando* XRD patterns in Fig. 3b, the peaks of the (010) plane at 8.25° and 16.74° gradually disappeared after the reaction with water, meaning that the chemical bonds between the Tb atoms and O atoms of DHBDC were broken and that coordination between Tb and water occurred. With an increase in water content of 25%, a characteristic amorphous peak at 27.4° appeared, indicating that the water molecules triggered the complete decomposition of the nanoprobe. This finding was also confirmed by the Tb4d XPS spectrum of the nanoprobe, in which the Tb^3+^ ions gave a signal shift from 152.60 eV to 151.04 eV (Fig. 3c). The shift of 1.56 eV to a lower binding energy may be due to the increase in electron density on the Tb atoms, caused by the coordination of the water molecules.^28^ In addition, a new band appeared at 534.9 eV in the O1s XPS spectrum of the nanoprobe, which was assigned to the chemisorbed water molecules.^29^ From the data for electrospray ionization mass spectrometry (ESI-MS), a single peak at *m*/*z* = 197 after the excess addition of water was consistent with that of the free DHDBC ligands, providing further information on the structural decomposition (Fig. S7 and Table S1†). Control experiments were conducted to further test the change in the FL spectra of DHBDC on the addition of successive aliquots of water. The presence of water caused a significant red-shift, in which the emission peak moved from 450 nm to 505 nm, and the intensity of the peak at 450 nm gradually decreased and the intensity of the peak at 505 nm increased (Fig. S8 and S9†). The results are consistent with the above responsive behavior of the nanoprobe, and they provide evidences for the hypothesis that the selectivity of the nanoprobe for water is due to the presence of dissociative DHBDC ligands. Therefore, all of the experimental results provide solid evidences for the reaction process in which the Tb atoms coordinate with water molecules followed by the dissociation of the DHBDC ligands.

Having established the ability of the nanoprobe to respond selectively to water molecules in organic solvents, we further explored its potential applications for the direct measurement of water content in Li-ion batteries. As we know, water has severe influences on battery performance and it even affects the battery state of health. In fact, many side reactions during the electrochemical cycles of batteries can produce water molecules as by-products. Among these side reactions, the solid electrolyte interface (SEI) layer has received much attention because of its outstanding performance.^30^ The SEI layer is formed on the electrode surface during the first electrochemical cycle and works as a passivation layer to block electron transport and allow Li^+^ ions to pass through, thereby resulting in the prevention of electrolyte degradation. Therefore, the SEI layer plays an important role in preserving the integrity of the electrodes, preventing side reactions between the solvents and electrodes and retaining a high reversible capacity and good cycle performance. Although the mechanism of the formation of the SEI layer is currently still unclear, it has been proven that trace water is generated as an inevitable by-product with a concentration as low as the ppm-level.^31^,^32^ Therefore the nanoprobe, in combination with *in situ* FL spectroscopy, will present the first example of the direct monitoring of water content in real-time in a Li-ion cell.

We first investigated the nanoprobe’s performance for the determination of water content in a commercial electrolyte, *i.e.* 1EC–1EMC–1DEC. The sensitivity was evaluated by monitoring the fluorescence changes with varying aliquots of water (0–1.2% v/v). As indicated in Fig. S10,† the fluorescence intensity at 450 nm increased linearly upon increasing the water concentration (*R*^2^ = 0.998), which was in accordance with the FL responsive behavior of the nanoprobe in MeCN. The above results prove that the as-prepared nanoprobe would be a promising candidate to monitor trace water in Li-ion batteries.

**Fig. 4:**
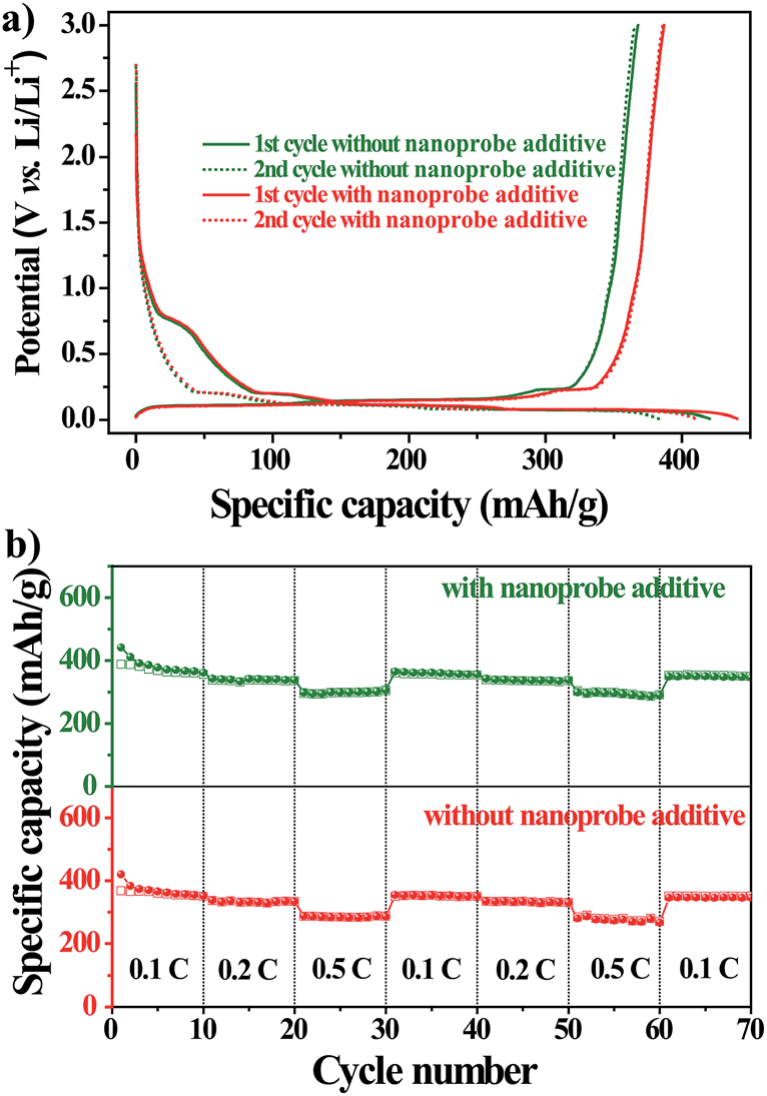
(a) The voltage–capacity profile of the first two charge/discharge cycles of the nanoprobe-free and nanoprobe-containing samples at a current of 0.1 C. (b) The specific capacity as a function of the cycle number for the nanoprobe-free and nanoprobe-containing samples at various current rates.

To further explore the practical applications, we tested the electrochemical performances of Li-ion batteries with the nanoprobe as an additive in a CR2032-type coin cell at 298 K. Fig. 4a shows the voltage–capacity profile of the first two charge/discharge cycles of the nanoprobe-free and nanoprobe-containing samples at a constant current of 0.1 C. For either sample, the profile exhibited a distinct plateau at 0.8 V *versus* Li/Li^+^, followed by a gradual slope from 0.8 V to 0.01 V. The total capacity for the first cycle reached 493 and 564 mA h g^–1^ for the nanoprobe-free and nanoprobe-containing samples, respectively. For the second electrochemical cycle, a reversible capacity of 450 mA h g^–1^ was recovered for the cell with the nanoprobe as an electrolyte additive, and only 398 mA h g^–1^ was recovered for the cell without the nanoprobe. The capacity loss of about 20% for either sample could be attributed to the formation of the SEI layer on the surface of the graphitic electrode, which was associated with LiPF_6_ decomposition and the formation of lithium compounds.^33^,^34^ The presence of the nanoprobe in the electrolyte increased the specific capacity by at least 10% compared to that obtained using the standard electrolyte.^35^,^36^ To further confirm that the presence of the nanoprobe has no noticeable influence, a study of cycle performance was conducted. Fig. 4b shows the specific capacity *versus* the cycle number for the nanoprobe-free and nanoprobe-containing samples at various current rates. After 50 cycles, the nanoprobe-containing cell maintained a stable capacity, revealing that the presence of the nanoprobe in the electrolyte has no pronounced negative effect on the cycle performance of Li-ion batteries, even at a higher current rate of 0.5 C. Based on the above electrochemical results, the nanoprobe as an additive in the electrolyte solution has no distinct influence on the performance of lithium-ion batteries, which satisfies the prerequisite condition for the direct monitoring of trace water in Li-ion batteries.

**Fig. 5:**
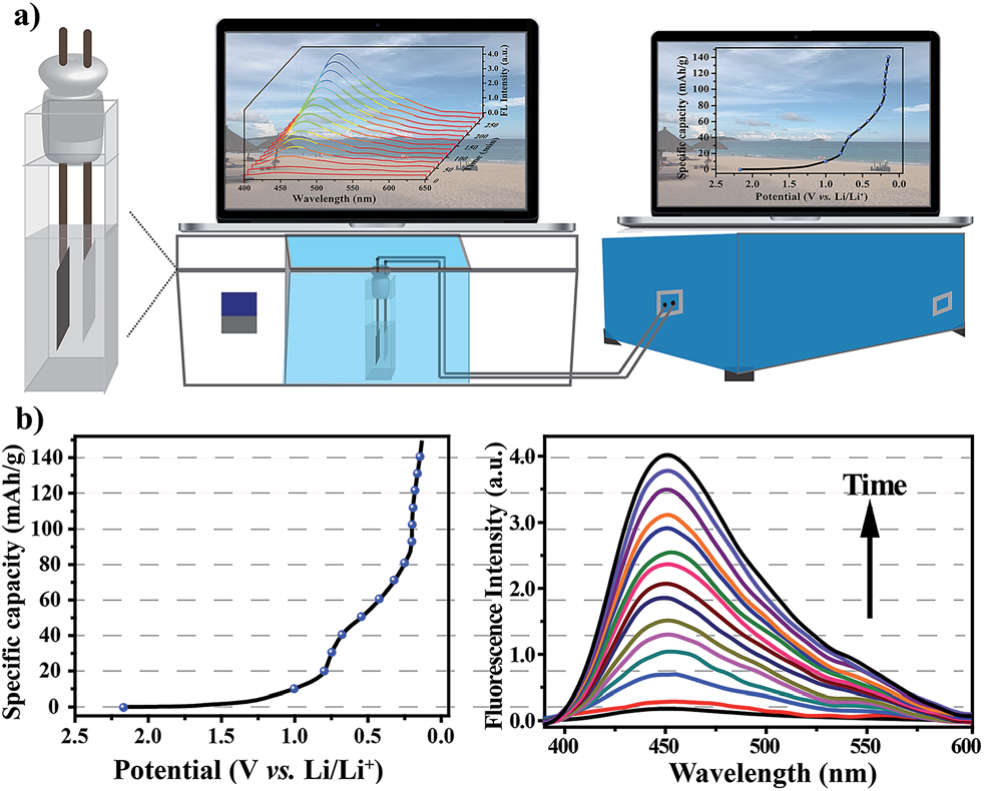
(a) A schematic diagram of the home-made *in situ* cell and measurement system. (b) Changes in the fluorescence spectra of the electrolyte with the nanoprobe as an electrolyte additive during the first discharge process.

After verifying that the nanoprobe was able to detect water in the commercial electrolyte, EC–EMC–DEC, and that the presence of the nanoprobe as an additive had no negative effect on battery performance, we next sought to explore its applicability to measure water content *in situ* during electrochemical cycles. To simplify the cell system, a proof-of-concept water-detection prototype was built to demonstrate the feasibility of this approach, as shown in Fig. 5a. An *in situ* cell was home-made with an external size of 2.2 × 2.2 × 4 cm^3^ and a suitable silica gel plug with two circular holes with diameters of 2 mm. A series of FL spectra were recorded, at a frequency of one pattern per 20 minutes, to track the dynamic processes during the first discharge process that was the formation period of the SEI layer (Fig. 5b). The intensity of the FL emission at 450 nm gradually increased over time, which clearly proved that the generation of water occurred. A linear correlation was found between the FL intensities and time (Fig. S11†), revealing that water was gradually generated and that its content linearly increased (*R*^2^ = 0.997).

When irradiated at 365 nm, the FL color of the electrolyte obviously changed from colorless to light blue after the first cycle (Fig. S13†). After the first discharge process, the intensity of the fluorescence emission was nearly invariable (Fig. S12†), revealing that the formation reaction of the SEI layer was the main side-reaction to generate water. From the fluorescence analysis, the amount of water generated was calculated to be about 0.18% in 2 mL electrolyte.

The mechanism involved in the formation of the SEI layer is far more complicated and is still unclear even today, but all experimental results confirm that water is definitely generated as a by-product during the first discharge process, and its content can be accurately calculated. The FL spectra reflect the change in water content, in which the FL intensity shows a linear increase over time along with the gradual generation of water, enabling *in situ* and real-time measurements of water content. Although this approach is operated under conditions that are far from realistic, the developed *in situ* cell and methodology offer an opportunity to track the chemical reaction and the formation of water during charge/discharge processes, for the first time, using coordination polymer nanoparticles as a water probe. This approach provides new insights into the *in situ* monitoring of complex electrochemical processes, and may help to pave the way for the development of new *operando* analytical techniques for transparent lithium-ion batteries.^37^

## Conclusions

The nanoprobe provides an effective platform for the super-selective detection of water molecules, and for the first time realizes the *in situ* measurement of water content in a Li-ion cell. The new method offers several advantages over traditional organic fluorescent molecules/quantum dots. Firstly, the synthesis method is facile and does not require expensive and complicated instruments. Secondly, the method allows the rapid, visible and “turn-on” detection of water molecules with a volumetric detection limit of 0.03%, to be achieved with the naked eye within 1 min. Thirdly, the nanoprobe exhibits excellent selectivity for water over other common organic solvents. These advantages make the nanoprobe very promising for the *in situ* tracking of trace water during electrochemical cycles. Based on the designed *in situ* measurement system, it is possible to obtain qualitative and quantitative measurements of water content during the first discharge process, in which the FL intensity shows a linear increase over time along with the gradual generation of water. Therefore, the nanosized coordination polymers, as a water nanoprobe with super sensitivity, in combination with *operando* fluorescence spectroscopy, provide an effective platform for the *in situ* and real-time monitoring of trace water in Li-ion batteries. This work provides a new strategy for tracking complex dynamic processes and a quantitative measurement of impurities in electrolytes, and it may help to pave the way for the development of novel *operando* analytical techniques for lithium-ion batteries.

## Conflicts of interest

There are no conflicts to declare.

## Supplementary Material

Supplementary information

## References

[cit1] Tarascon J. M., Armand M. (2001). Nature.

[cit2] Goodenough J. B., Park K. S. (2013). J. Am. Chem. Soc..

[cit3] Lu L., Han X., Li J., Hua J., Ouyang M. (2013). J. Power Sources.

[cit4] Wang J., Zhang Y., Guo L., Wang E., Peng Z. (2016). Angew. Chem., Int. Ed..

[cit5] Emanuel P. and Diana G., Lithium-ion batteries: solid-electrolyte interphase, Imperial College Press, London, 2004.

[cit6] Li Y., Leung K., Qi Y. (2016). Acc. Chem. Res..

[cit7] Zhuang G., Ross P. N., Kong F., McLarnon F. (1998). J. Electrochem. Soc..

[cit8] Ross P. N. (2014). Catal. Lett..

[cit9] Aurbacha D., Weissmana I., Zabana A., Dan P. (1999). Electrochim. Acta.

[cit10] Shi F., Ross P. N., Zhao H., Liu G., Somorjai G. A., Komvopoulos K. (2015). J. Am. Chem. Soc..

[cit11] Arora P., White R. E., Doyle M. (1998). J. Electrochem. Soc..

[cit12] Xu K. (2004). Chem. Rev..

[cit13] Sherman F. B. (1980). Talanta.

[cit14] Ogata K., Salager E., Kerr C. J., Fraser A. E., Ducati C., Morris A. J., Hofmannm S., Grey C. P. (2014). Nat. Commun..

[cit15] Key B., Bhattacharyya R., Morcrette M., Seznéc V., Tarascon J. M., Grey C. P. (2009). J. Am. Chem. Soc..

[cit16] Sathiya M., Leriche J.-B., Salager E., Gourier D., Tarascon J. M., Vezin H. (2015). Nat. Commun..

[cit17] Wang F., Yu H.-C., Chen M.-H., Wu L., Pereira N., Thornton K., Van der Ven A., Zhu Y., Amatucci G. G., Graetz J. (2012). Nat. Commun..

[cit18] Liu H., Strobridge F. C., Borkiewicz O. J., Wiaderek K. M., Chapman K. W., Chupas P. J., Grey C. P. (2014). Science.

[cit19] Grey C. P., Tarascon J. M. (2017). Nat. Mater..

[cit20] Mai L., Yan M., Zhao Y. (2017). Nature.

[cit21] Ma X., Luo W., Yan M., He L., Mai L. (2016). Nano Energy.

[cit22] de Silva A. P., Gunaratne H. Q. N., Gunnlaugsson T., Huxley A. J. M., McCoy C. P., Rademacher J. T., Rice T. E. (1997). Chem. Rev..

[cit23] Cui Y., Yue Y., Qian G., Chen B. (2012). Chem. Rev..

[cit24] Park D.-H., Park B. J., Kim J.-M. (2016). Acc. Chem. Res..

[cit25] Lou Q., Qu S., Jing P., Ji W., Li D., Cao J., Zhang H., Liu L., Zhao J., Shen D. (2015). Adv. Mater..

[cit26] Wang Y.-L., Jiang Y.-L., Liu Q.-Y., Tan Y.-X., Wei J.-J., Zhang J. (2011). CrystEngComm.

[cit27] Douvali A., Tsipis A. C., Eliseeva S. V., Petoud S., Papaefstathiou G. S., Malliakas C. D., Papadas I., Armatas G. S., Margiolaki I., Kanatzidis M. G., Lazarides T., Manos M. J. (2015). Angew. Chem., Int. Ed..

[cit28] Sarma D. D., Rao C. N. (1980). J. Electron Spectrosc. Relat. Phenom..

[cit29] Kerber S. J., Bruckner J. J., Wozniak K., Seal S., Hardcastle S., Barr T. L. (1996). J. Vac. Sci. Technol., A.

[cit30] Xu K. (2014). Chem. Rev..

[cit31] Egashira M., Izumi T., Yoshimoto N., Morita M. (2016). J. Power Sources.

[cit32] Kwabi D. G., Batcho T. P., Feng S., Giordano L., Thompson C. V., Shao-Horn Y. (2016). Phys. Chem. Chem. Phys..

[cit33] Chan C. K., Zhang X. F., Cui Y. (2008). Nano Lett..

[cit34] Ban C., Wu Z., Gillaspie D. T., Chen L., Yan Y., Blackburn J. L., Dillon A. C. (2010). Adv. Mater..

[cit35] Meng J., Niu C., Xu L., Li J., Liu X., Wang X., Wu Y., Xu X., Chen W., Li Q., Zhu Z., Zhao D., Mai L. (2017). J. Am. Chem. Soc..

[cit36] Balogun M., Wu Z., Luo Y., Qiu W., Fan X., Long B., Huang M., Liu P., Tong Y. (2016). J. Power Sources.

[cit37] Yang Y., Jeong S., Hu L., Wu H., Lee S. W., Cui Y. (2011). Proc. Natl. Acad. Sci. U. S. A..

