# Supplementary material for: Direct monitoring of trace water in Li-ion batteries using operando fluorescence spectroscopy
Source: Chem Sci. 2017 Oct 23;9(1):231–7. doi: 10.1039/c7sc03191b (PMC5869306; doi:10.1039/c7sc03191b)
Supplement: Supplementary file 1 [file SC-009-C7SC03191B-s001.pdf]

Electronic Supplementary Material (ESI) for Chemical Science.
This journal is © The Royal Society of Chemistry 2017

Supporting Information

Direct monitoring of trace water in Li-ion batteries using *operando* fluorescence spectroscopy

Xiaoyan Ren, Jiawei Wang, Zhangquan Peng* and Lehui Lu*

State Key Laboratory of Electroanalytical Chemistry, Changchun Institute of Applied Chemistry, Chinese Academy of Sciences, Changchun 130022, China.

E-mail: zqpeng@ciac.ac.cn, lehuilu@ciac.ac.cn

Experimental

Materials and reagents

2,5-dihydroxyterephthalic acid (DHBDC), acetonitrile (MeCN), dimethyl formamide (DMF), and anhydrous terbium chloride were purchased from Sinopharm Chemical Reagent Co., Ltd. SFG-15 graphite powder (Timcal Co. Ltd.) and polytetrafluoroethylene (PTFE, Aldrich) were used as received. All organic solvents and electrolytes including ethyl methyl carbonate (EMC), ethylene carbonate (EC), and diethyl carbonate (DEC) were of analytical grade and dried by 4 Å molecular sieves (NaA zeolite) until the H_2O concentration was lower than 10 *ppm* that was determined by a Mettler-Toledo Karl-Fischer titration apparatus.

Synthetic procedures

Preparation of bulk crystals. A mixture of $TbCl_3$ (0.135 g) and 2,5-dihydroxy-terephthalic acid (DHBDC, 0.099 g) were dissolved in 10 mL DMF, and then loaded into a stainless steel vessel (20 mL). The vessel was sealed and heated to 60 ℃ for two days. After the reaction mixture was cooling down to room temperature, plate-like crystals were obtained, which were further washed by using DMF for three times.

Preparation of nanoprobe. Nanosized product was successfully synthesized in large-scale *via* a nano-precipitation process. $TbCl_3$ (26.5 mg) was mixed with 2,5-dihydroxyterephthalic acid (DHBDC, 20.0 mg) in DMF (5.0 mL) solution and then heated at 60 ℃ for two hours. After that MeCN was added quickly to form the nanosized precipitate (V_{DMF}/V_{MeCN} = 1 : 2). The precipitate was collected by centrifugation, washed by MeCN for several times, and then redispersed in MeCN.

Instrumentations

Fluorescence emission spectra were taken using an F-4500 spectrophotometer with a 150W xenon lamp. Scanning electron microscopy (SEM) images were carried out by using a FEI/Philips XL30 ESEM FEG field-emission scanning

electron microscope under an acceleration voltage of 20 kV. X-ray diffraction (XRD) patterns were acquired by using a D8ADVANCE (Germany) with Cu-Ka radiation (λ = 1.5406 nm). Thermogravimetric analysis (TGA) was obtained by a Perkin-Elmer TGA-2 thermogravimetric analyzer and the samples were heated in air from room temperature to 1000 ℃ at a heating rate of 10 ℃/min. UV/vis adsorption spectra were collected by a VARIAN CARY 50 UV/vis spectrophotometer. Fourier transform infrared spectroscopy (FTIR) spetrum was obtained on a Bruker Vertex 70 spectrometer in the 400-4000 cm^{-1} region. The element analyses were carried out by using a Perkin-Elmer 2400 elemental analyzer. Single-crystal X-ray diffraction data was obtained on a Bruker AXS SMART APEX-II diffractometer with Mo-Kα (λ = 0.71073 Å) radiation at 298 K. Adsorption/desorption isotherm was measured using CO_2 gas at 298 K on Micromeritics ASAP 2020. X-ray photoelectron spectroscopy (XPS) was acquired by using a VGESCALAB MKII spectrometer. The XPS data was analysed by the XPSPEAK software to deconvolute the narrow-scan XPS spectra of the sample. Electrospray ionization mass spectrometry (ESI-MS) data were obtained on a LTQ linear ion trap mass spectrometer with a conventional ESI source (Thermo, San Jose, CA, USA).

Electrochemical Characterization

In order to realize the simultaneous analysis of electrochemical properties and fluorescence spectra, an *in-situ* cell was home-made with external sizes of 2.2*2.2*4 cm^3 and a suitable silica-gel plug with two circular holes with diameters of 2 mm. The composite carbon electrodes were obtained by mixing 90 *wt*. % SFG-15 graphite and 10 *wt*. % polytetrafluoroethylene in 2-propanol to form uniform slurries, which were then coated onto a stainless steel mesh current collector. The prepared electrodes were dried under vacuum at 60 ℃ overnight. The loaded active material was about 2-3 mg/cm^2 in the experimental conditions. As for counter/reference electrode, a slice of Lithium foil was pressed on a stainless steel mesh current collector. The electrolyte was

1 M $LiPF_6$ in a mixture of 1 EC: 1EMC: 1DEC. Stock electrolyte solutions were prepared by dispersing freeze-drying nanoprobe in above electrolyte with the concentration of 10 μg/mL. A 2-electrodes cell consisted of a thin Li foil as the counter/reference electrode and composite carbon electrode as working electrode. The cell was assembled in an argon-filled glove box and finally sealed by using parafilm to avoid contamination by moisture and oxygen. The electrochemical tests were collected on a Parstat 4000 electrochemical workstation with the voltage range of 0.01-3.0 V *vs.* Li^+/Li. CR2032-type Coin-cells were assembled by using similar composite as electrodes and sealed in an argon-filled glove box to avoid external contamination. The electrochemical cycles were taken on a LANHE CT2001C multi-channel battery testing system with the voltage range of 0.01-3.0 V *vs*. Li^+/Li.

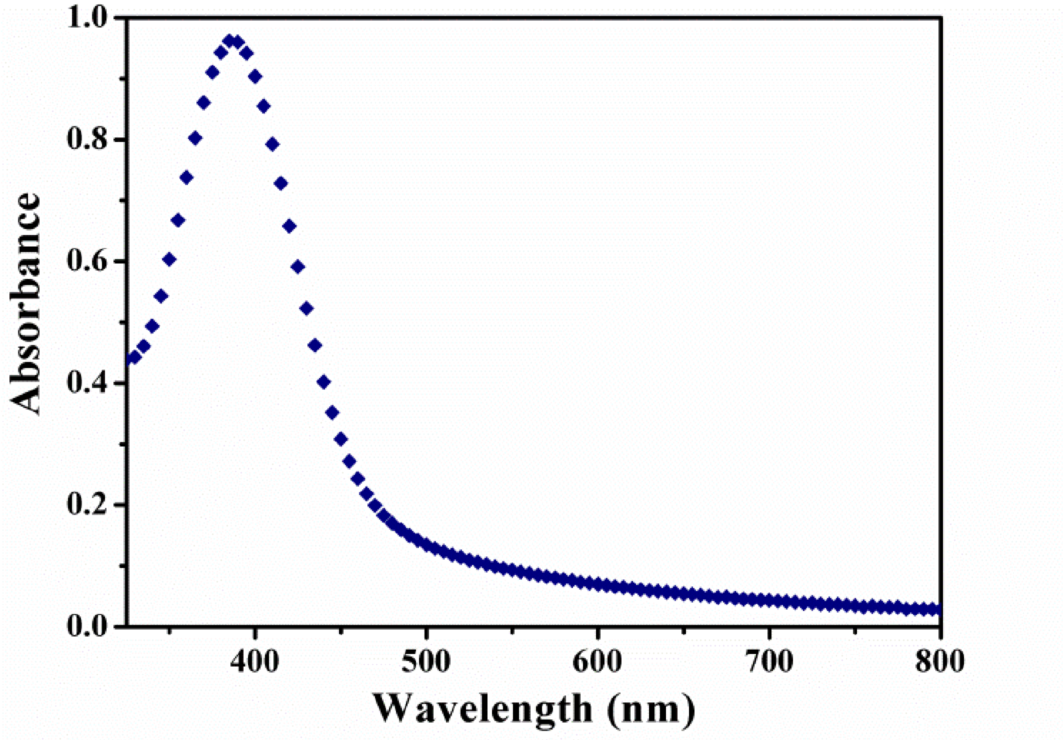

Fig.S1 Adsorption spectra of nanoprobe suspension. The UV-vis spectrum of nanoprobe indicated a broad adsorption centered around 385 nm.

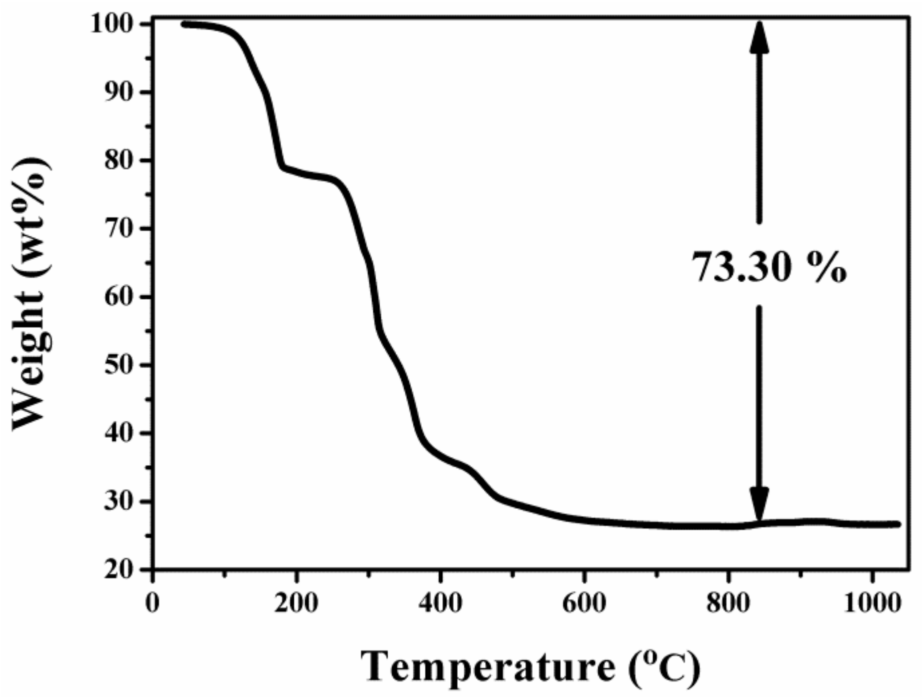

Fig.S2 TGA result of the nanoprobe.

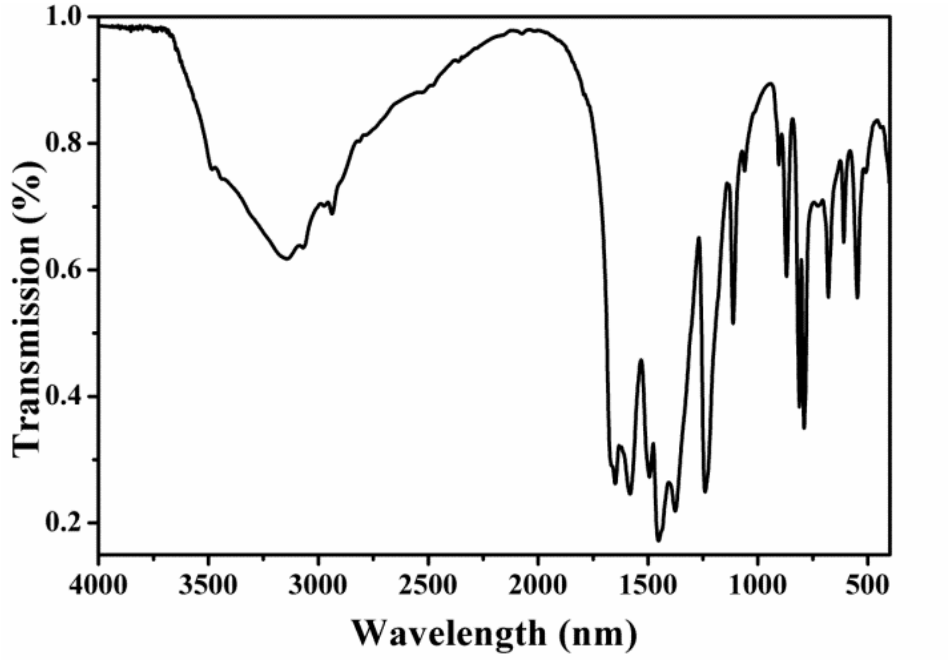

Fig.S3 FTIR analysis of the nanoprobe.

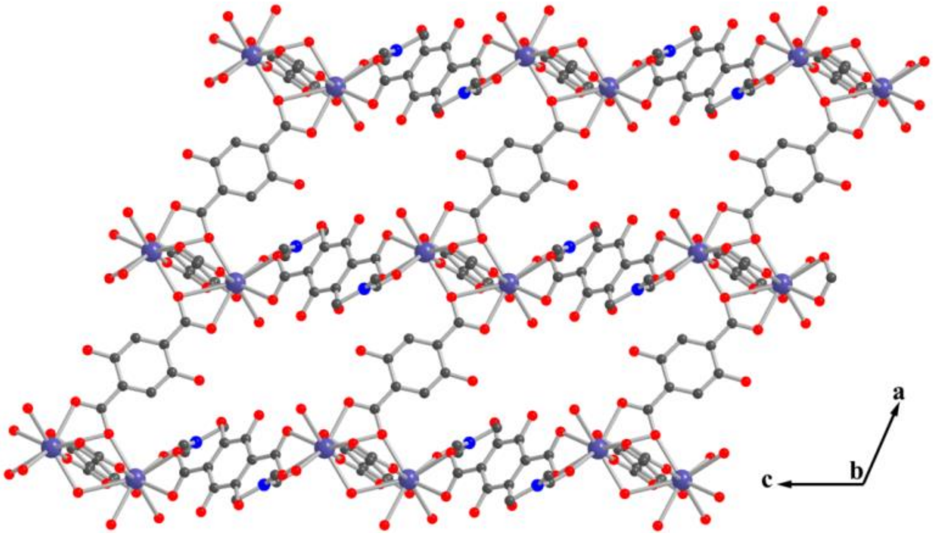

Fig. S4 The framework of nanoprobe viewed along *b* axis (Tb, purple; C, gray; O, red).

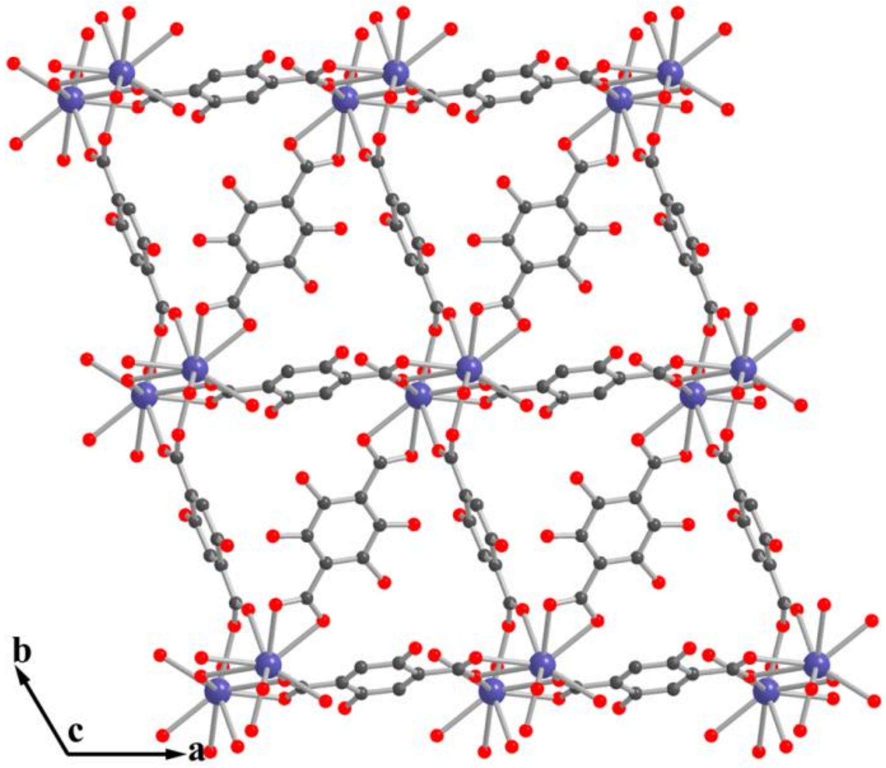

Fig. S5 The framework of nanoprobe viewed along *c* axis (Tb, purple; C, gray; O, red).

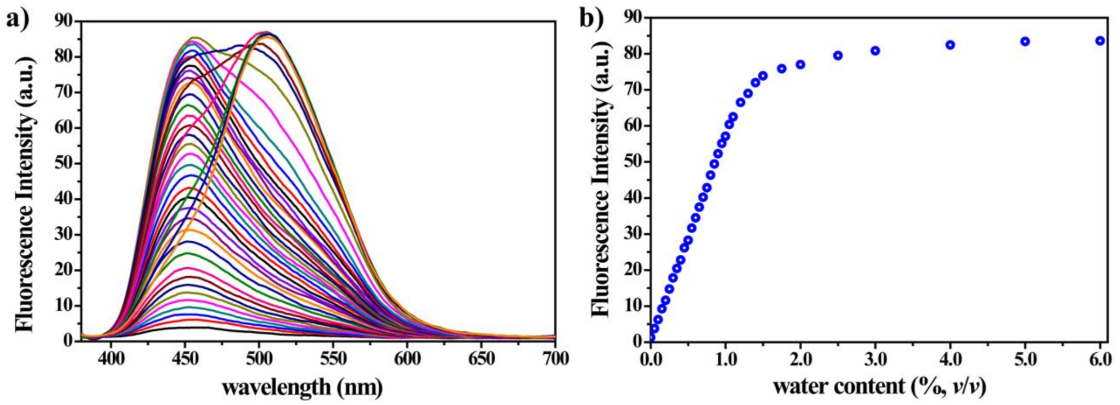

Fig. S6 Changes in the fluorescence spectrum of nanoprobe suspension in dry MeCN in the presence of different aliquots of water (0–6% *v/v*). The excitation wavelength was λ_{ex} = 360 nm; (b) the plot of fluorescence intensity as a function of water content.

Table S1. The element contents of Tb, C, O changed with the water content.

H_2O (*v*/*v*)	Tb	C	O
0	25.53	38.48	35.99
1 %	24.04	39.02	36.94
5 %	21.50	41.90	36.61
10 %	18.14	43.77	38.09
25 %	16.17	47.94	35.88
50 %	14.56	46.59	38.85

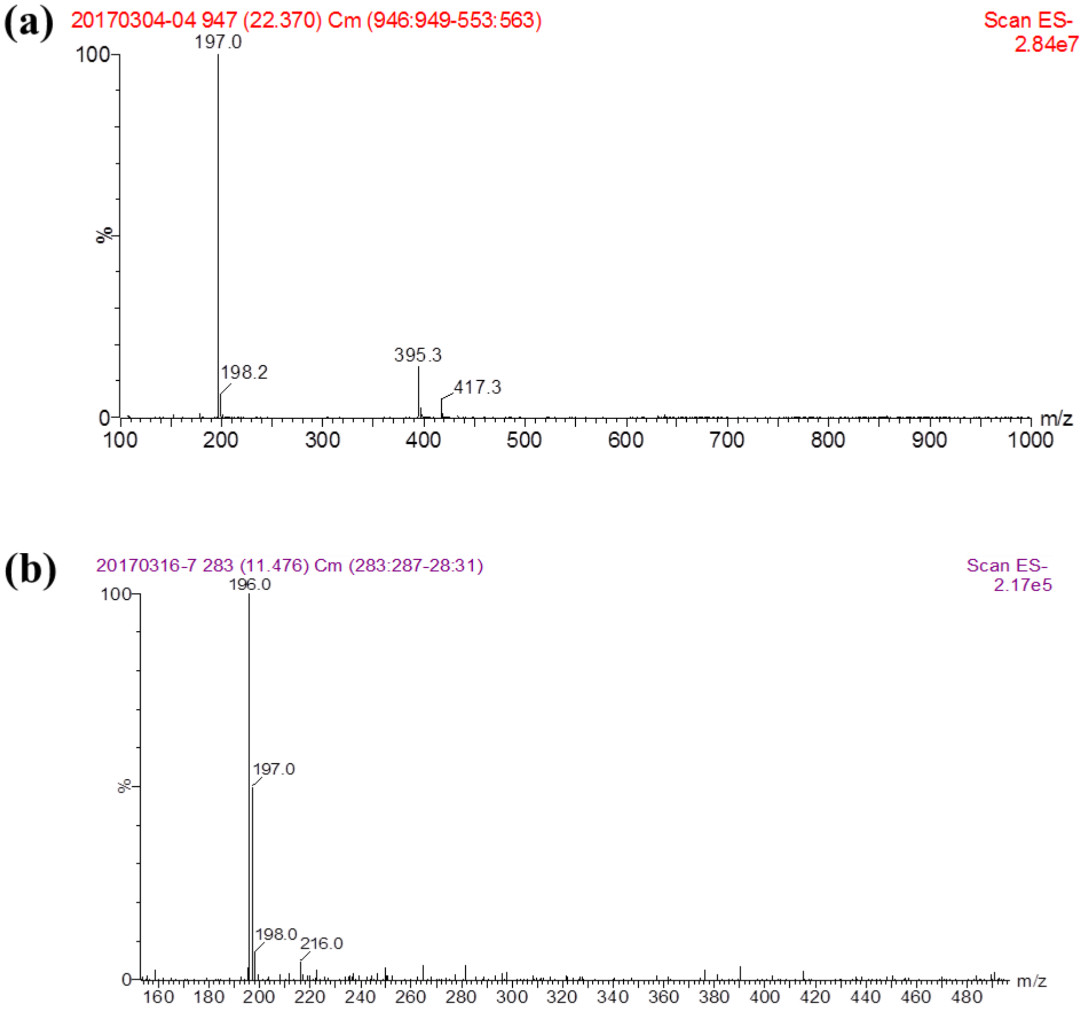

Fig.S7 Massspectrum of (a) free DHDBC ligands; (b) nanoprobe with excess water.

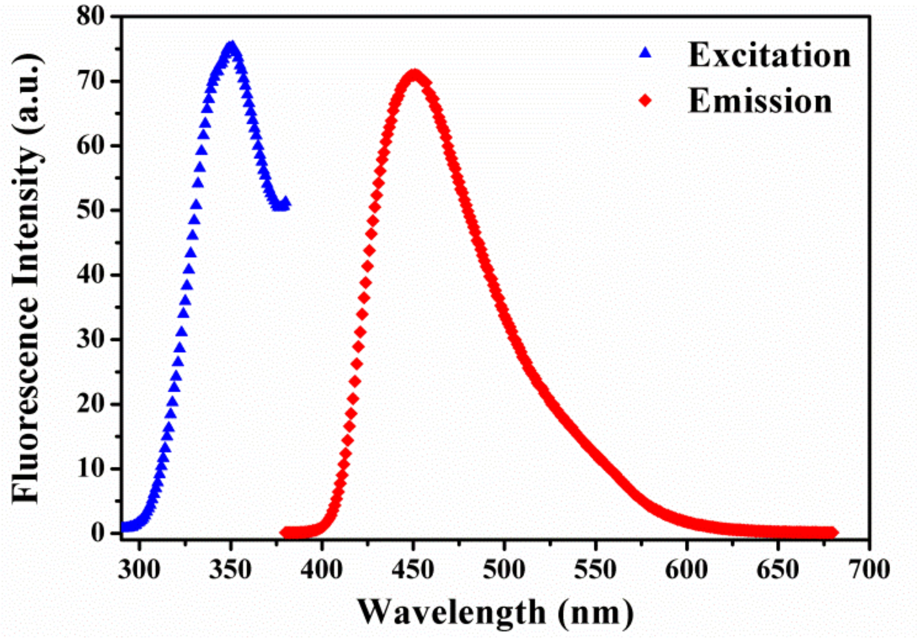

Fig.S8 Fluorescence excitation and emission spectra of DHBDC.

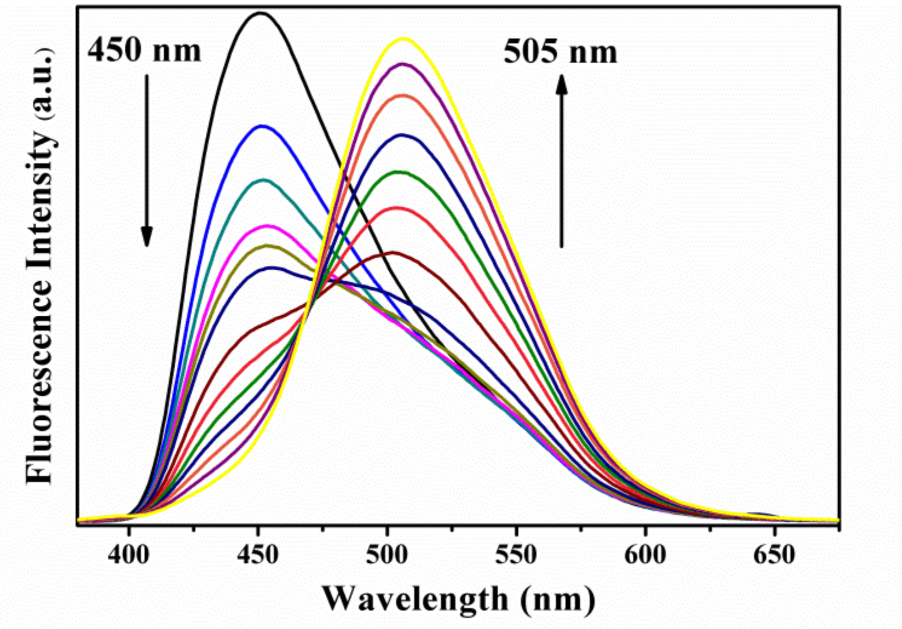

Fig. S9 Change of emission spectra of DHBDC in the presence of different aliquots of water, such as 0, 0.25, 0.5, 0.75, 1.0, 1.5, 2.0, 2.5, 3.0, 3.5, 4.0, 4.5, 5.0 %*v*/*v*.

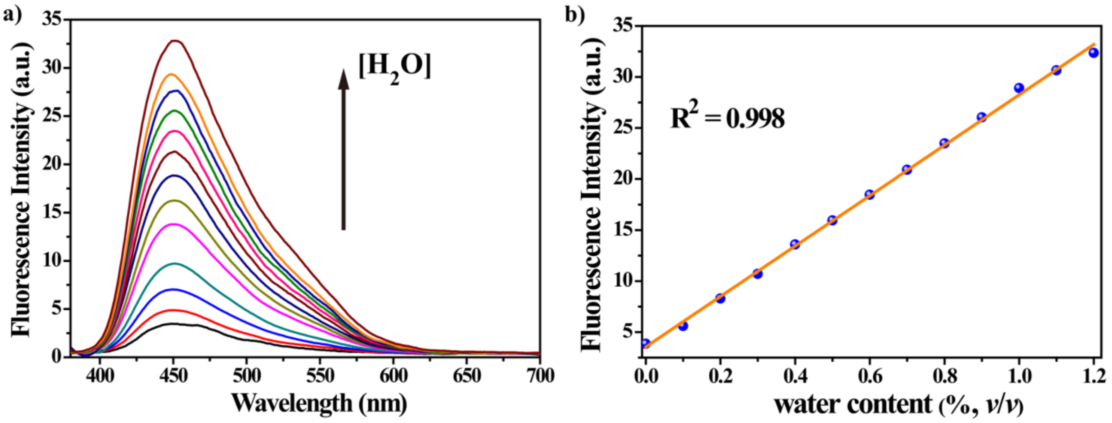

Fig. S10 a) Changes in the fluorescence spectrum of nanoprobe suspension in commercial EC-EMC-DEC in the presence of different aliquots of water (0–1.2 % *v*/*v*). The excitation wavelength was λ_{ex} = 360 nm. b)The plot of fluorescence intensity as a function of water content.

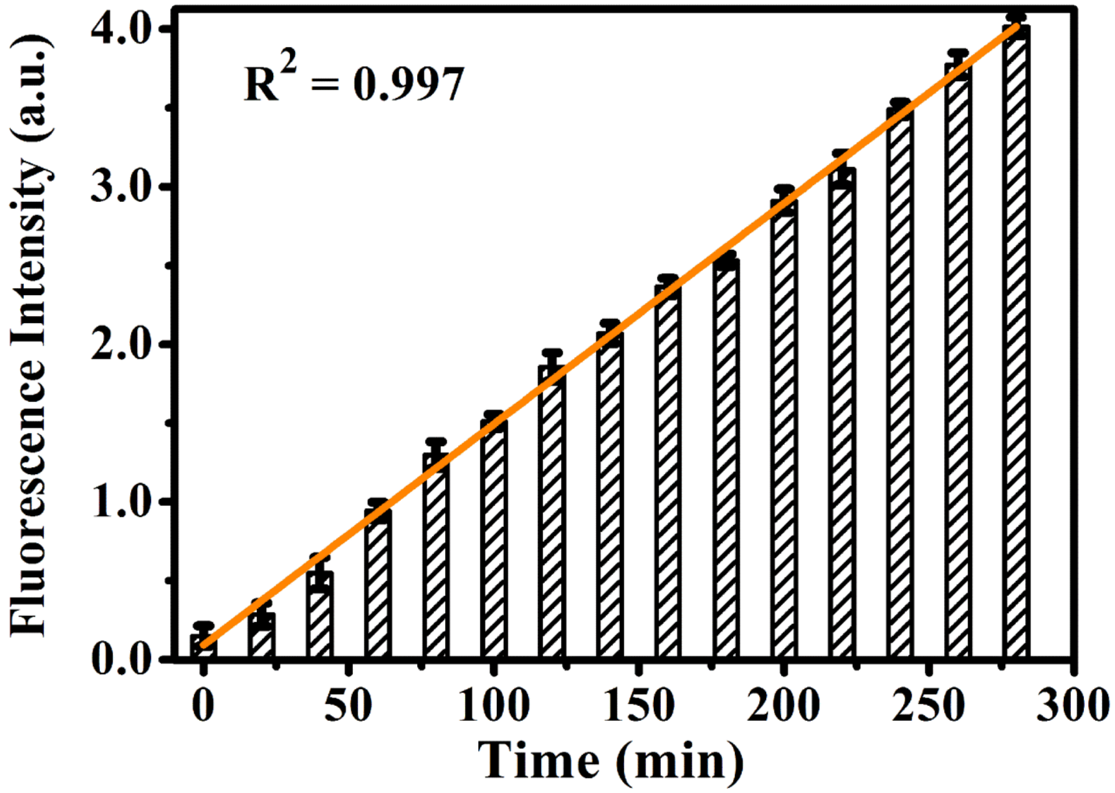

Fig. S11 Plot of fluorescence intensity as a function of reaction time during the formation process of SEI layer.The error bars indicate standard deviation from three measurements.

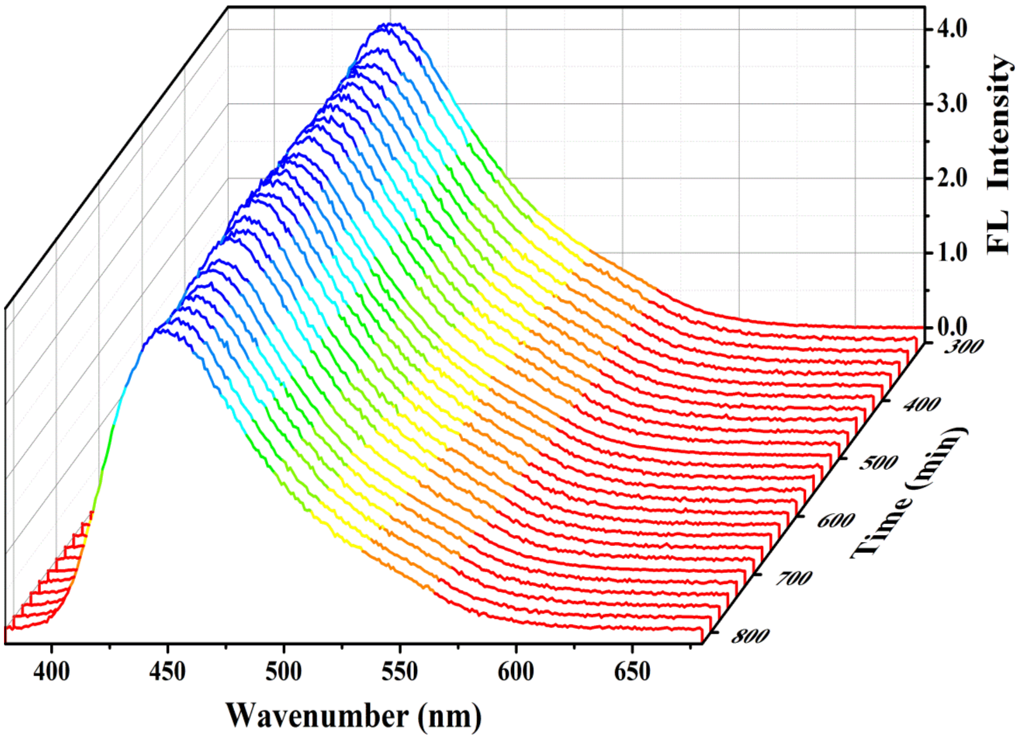

Fig. S12 Changes in the fluorescence spectrum of nanoprobe suspension are nearly invariable after the formation process of SEI layer.

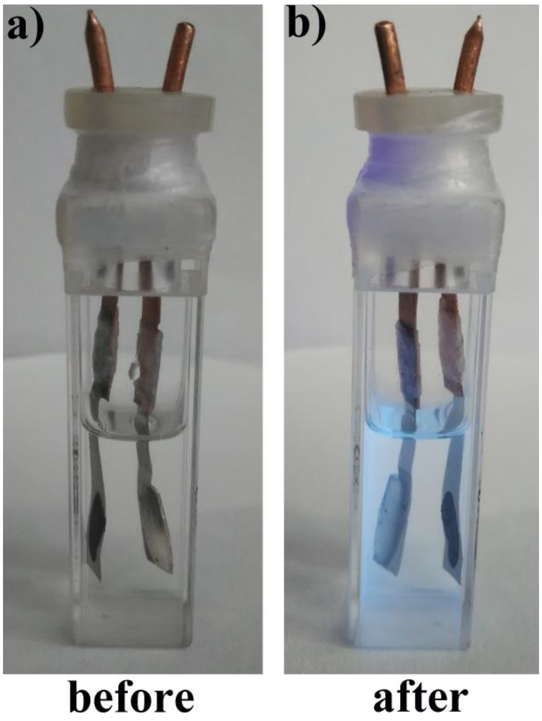

Fig. S13 The fluorescence color of electrolyteis obviously changedfrom colorless to light blue after the first cycle, when irradiated with a UV hand lamp at λ_{ex} = 365 nm.
